# [Nle^4^, D-Phe^7^]-α-MSH Inhibits Toll-Like Receptor (TLR)2- and TLR4-Induced Microglial Activation and Promotes a M2-Like Phenotype

**DOI:** 10.1371/journal.pone.0158564

**Published:** 2016-06-30

**Authors:** Lila Carniglia, Delia Ramírez, Daniela Durand, Julieta Saba, Carla Caruso, Mercedes Lasaga

**Affiliations:** Institute of Biomedical Research (INBIOMED -UBA- CONICET), School of Medicine, University of Buenos Aires, Buenos Aires, 1121, Argentina; National Institute of Allergy and Infectious Diseases - Rocky Mountain Laboratories, UNITED STATES

## Abstract

α-melanocyte stimulating hormone (α-MSH) is an anti-inflammatory peptide, proved to be beneficial in many neuroinflammatory disorders acting through melanocortin receptor 4 (MC4R). We previously determined that rat microglial cells express MC4R and that NDP-MSH, an analog of α-MSH, induces PPAR-γ expression and IL-10 release in these cells. Given the great importance of modulation of glial activation in neuroinflammatory disorders, we tested the ability of NDP-MSH to shape microglial phenotype and to modulate Toll-like receptor (TLR)-mediated inflammatory responses. Primary rat cultured microglia were stimulated with NDP-MSH followed by the TLR2 agonist Pam_3_CSK_4_ or the TLR4 agonist LPS. NDP-MSH alone induced expression of the M2a/M2c marker *Ag1* and reduced expression of the M2b marker *Il-4rα* and of the LPS receptor *Tlr4*. Nuclear translocation of NF-κB subunits p65 and c-Rel was induced by LPS and these effects were partially prevented by NDP-MSH. NDP-MSH reduced LPS- and Pam_3_CSK_4_-induced TNF-α release but did not affect TLR-induced IL-10 release. Also, NDP-MSH inhibited TLR2-induced HMGB1 translocation from nucleus to cytoplasm and TLR2-induced phagocytic activity. Our data show that NDP-MSH inhibits TLR2- and TLR4-mediated proinflammatory mechanisms and promotes microglial M2-like polarization, supporting melanocortins as useful tools for shaping microglial activation towards an alternative immunomodulatory phenotype.

## Introduction

Microglial cells are key players in the inflammatory processes within the central nervous system (CNS). They are in charge of immune surveillance and respond to injury or noxious stimuli by releasing inflammatory mediators, recruiting other immune cells to the injured zone and orchestrating an effective inflammatory response. Afterwards, they release anti-inflammatory mediators leading to resolution of the inflammatory response and return to homeostasis. Alterations in this delicate process may lead to tissue damage, neuroinflammation and neurodegeneration. Microglia can be found in two main activation states, termed M1 and M2. M1 microglia display a classical pro-inflammatory program which can be induced by T_H_1 cytokines such as IFN-γ or bacterial moieties such as lipopolysaccharide (LPS), and is characterized by the production of tumour necrosis factor (TNF)-α, Interleukin (IL)-1β, IL-12, reactive oxygen species (ROS) and nitric oxide (NO), among other mediators. M2, in turn, refers to alternatively activated microglia, a phenotype induced by T_H_2 cytokines such as IL-4 and IL-13, whose main markers include Arginase-1 (AG1), IL-10, transforming growth factor (TGF)-β and peroxisome proliferator-activated receptor (PPAR)-γ, among others [1].

As immunocompetent cells, microglia express a variety of innate immunity receptors, such as Toll-like receptors (TLRs) [2]. TLRs comprise a family of membrane-associated receptors that bind a diversity of pathogen-associated molecular patterns (PAMPs) [3]. Alternatively, TLRs can be activated by the presence of endogenous molecules released after tissue damage, termed damage associated molecular patterns (DAMPs) [4]. In particular, TLR2 and TLR4 can mediate the actions of the DAMP high-mobility group box 1 (HMGB1), which can be released to the extracellular milieu during traumatic cell death or upon stimulation by a number of inflammatory mediators such as TLR agonists, and potentiate the inflammatory response [4, 5]. Plus, activation of microglial TLR2 and TLR4 has been associated with neurotoxicity in various CNS diseases [6], further underscoring the importance of studying the modulation of TLR signaling in these cells. The triacylated lipoprotein Pam_3_CSK_4_ and LPS are frequently used as TLR2 and TLR4 agonists, respectively [7]. The binding of Pam_3_CSK_4_ or LPS to their receptors leads to the activation of the nuclear transcription factor NF-κB. NF-κB family is composed of five subunits, p65/RelA, p50, p52, c-Rel and RelB, which can form homo or heterodimers. The C-terminal transcription activation domain, necessary for positive regulation of target genes, is present in p65, c-Rel and RelB but not in p50 and p52. Therefore, p50/p50 and p52/p52 homodimers are considered repressors of transcription [8]. The p50/p65 heterodimers are activated in most cell types whereas c-Rel-containing dimers are mainly activated in cells of the hematopoietic lineage [9]. Once activated, NF-κB translocates to the nucleus and promotes the expression of its target genes, such as TNF-α [10]. This cytokine in turn promotes and amplifies the inflammatory response. The release of inflammatory mediators is followed by production of immunomodulatory factors such as IL-10, a key player in resolution of the immune response [11]. When the inflammatory response is not properly resolved this otherwise necessary and beneficial process may turn against the organism and contribute to the development of neuroinflammatory disorders in the CNS. Therefore, identifying modulators of microglial activation is of paramount importance in the context of neuroinflammation and neurodegeneration.

α-melanocyte-stimulating hormone (α-MSH) is a member of the melanocortin family, a group of peptides derived from post-translational cleavage of pro-opiomelanocortin (POMC). Melanocortins exert their action through the activation of five melanocortin receptor subtypes (MC1R to MC5R) coupled to G-protein which upon activation induce production of cyclic AMP (cAMP) [12]. In the CNS melanocortins have proven to be anti-inflammatory and neuroprotective peptides in models of brain ischemia [13, 14] and sepsis [15] and to improve cognitive function, acting through MC4R, in a model of Alzheimer’s disease (AD) [16] or after cytokine treatment [17–19]. In a microglial cell line α-MSH was shown to inhibit the production of TNF-α, IL-6 and nitric oxide induced by LPS+IFN-γ [20]. We previously showed that rat cultured microglial cells express MC4R and that the synthetic peptide [Nle^4^, D-Phe^7^]-α-MSH (hereon NDP-MSH), an analog of endogenous α-MSH, induces PPAR-γ expression and IL-10 release from microglia, thereby promoting development of an anti-inflammatory phenotype [21]. Given the importance of modulating glial activation in the context of neuroinflammatory processes, this study tested the ability of NDP-MSH to modulate inflammatory mechanisms triggered by TLR2 and TLR4 agonists in microglial cells.

## Materials and Methods

### Reagents

NDP-MSH was purchased from Bachem (Bubendorf, Switzerland). Ultrapure LPS (E. coli, 0111:B4) and Pam_3_CSK_4_ were purchased from Invivogen (San Diego, CA, USA). Fluorescent microspheres (Molecular Probes, F8823) were kindly provided by Dr. Candolfi (INBIOMED, UBA-CONICET). Foetal bovine serum (FBS) was obtained from Natocor (Córdoba, Argentina). DMEM/F-12, DMEM, L-Glutamine and antibiotics were purchased from Invitrogen Life technologies (CA, USA). All other media and supplements were obtained from Sigma-Aldrich Corporation, unless specified otherwise.

### Primary microglial cultures

Wistar rats were bred in the INBIOMED animal housing facility, University of Buenos Aires, Buenos Aires, Argentina. Rats were kept in a 12 h light-dark cycle at 22°C ± 1°C with access to food and water *ad libitum*. 1–2 day-old Wistar rat pups were decapitated and their brains removed and freed from meninges. Cells were mechanically dispersed and seeded in previously poly-L-lysine-coated culture flasks, and held in DMEM/F-12 supplemented with 10% FBS, 50 μg/ml streptomycin and 50U penicillin, at 37°C in 5% CO_2_. Medium was replaced twice a week. After 11–14 days, microglial cells were detached from astrocytes by shaking for 1–2 h at 110 *rpm*, at 33°C in a Thermo Scientific Orbital Shaker (Thermo Fischer Scientific, Germany). Supernatants were collected and centrifuged, and microglia was seeded in uncoated plates in supplemented DMEM containing 10% FBS and 2 mM L-glutamine and left to stabilize overnight at 37°C in a 5% CO_2_ atmosphere before adding the drugs in fresh supplemented DMEM containing 2% FBS and 2 mM L-glutamine. Cells were stained with a microglial marker (anti-rat CD11b monoclonal antibody, OX-42, Millipore) to assess their purity, which was routinely close to 98%.

### Reverse-transcription real-time quantitative polymerase chain reaction (RT-qPCR)

Total RNA was extracted using Quickzol reagent (Kalium Technologies), following manufacturer’s instructions. 0.5–1 μg of RNA was treated with 1U DNAse (Promega Corp., Madison, WI) and reverse-transcribed as described before [22]. Products of the RT reaction were amplified using specific primers and SYBR Green Master Mix or SYBR Green Select Master Mix (Invitrogen Life Technologies) on a StepOne^™^ Real-Time PCR System (Applied Biosystems). Primer sequences are detailed in Table 1. PCR product specificity was verified by a melting curve analysis. No-RT controls were performed by omitting addition of the reverse transcriptase enzyme in the RT reaction, and no-template controls were performed by addition of nuclease-free water instead of cDNA. Gene expression was normalized to the endogenous reference gene HPRT by the ΔΔCt method [23] using Step-One Software (Applied Biosystems), and expressed as fold-changes relative to the control group.

**Table 1:** Primer sequences used for RT-qPCR.

**Gene**	Forward primer sequence	Reverse primer sequence
**AG1**	5’-GGAACCCTGGATGAGCATGA-3’	5’-AAAGGCGCTCCGATAATCTCT-3’
**SOCS3**	5’-AAAAATCCAGCCCCAATGGT -3’	5’-GGCCTGAGGAAGAAGCCTATC -3’
**IL-4Rα**	5’-TTCCAGAACCCTGTTCCTAACC-3’	5’-CTCCATGTCCAGTCCGAAGGT-3’
**HMGB1**	5’-CCCAAAAGCGTGAGCTTAAAA-3’	5’-ATGATAGCCTTCGCTGGGACTA-3’
**TLR4**	5’- CATTTACAGTTCGTCATGCTTTCTC-3’	5’- AGGCATCATCCTGGCATTTT-3’
**TLR2**	5’- CCCTGGAGGTGTTGGATGTTAG-3’	5’- AGCCGAGGCAAAAACAAAGA-3’
**HPRT**	5´-CTCATGGACTGATTATGGACAGGAC-3´	5´-CAGGTCAGCAAAGAACTTATAGCC-3´

Primer sequences for RT-qPCR reactions.

### Cell viability

Cells were treated with or without 100 nM NDP-MSH for 24 hours, then incubated with or without LPS or Pam_3_CSK_4_ (100 ng/ml) for 4.5 or 24 hours. Cell viability was assessed by 3-[4, 5-dimethylthiazol-2-yl]-2, 5-diphenyltetrazolium bromide (MTT) reduction assay. Briefly, cells were washed in Krebs buffer and incubated for 4 hours with 110 μl of a 0.5 μg/ml MTT-Krebs buffer solution. Formazan crystals obtained from MTT reduction were dissolved in 100 μl of a 0.04 N HCl-isopropanol solution. OD was measured in a microplate spectrophotometer (Bio-Rad) at 595 nm.

### Cytokine release

TNF-α, IL-10 and IL-4 release were assessed by ELISA using commercial kits (BD Biosciences, San Diego, USA). Assays were performed following the manufacturer’s instructions. Cytokine values (pg/ml) were normalized to the viability values obtained by the MTT reduction assay to account for variations in cell numbers caused by the different treatments.

### Immunocytochemistry

Cells were washed in PBS and fixed in 4% PBS–PFA for 10–15 minutes at RT. For p65 and c-Rel immunocytochemistry cells were incubated in ice cold methanol for 10 minutes at -20°C. For HMGB1 staining slides were incubated in Citrate buffer and subjected to microwave heating at 350 W for 5 minutes, then left to reach RT for 20 minutes and washed in PBS. Slides were incubated in blocking solution containing 10% donkey serum or 10% goat serum (depending on the source of the secondary antibody) in PBS for 1 hour at RT. Subsequently, cells were incubated overnight at 4°C with the following primary antibodies: anti-CD11b 1:100 (OX-42, Millipore), anti-p65 1:30 (BD Biosciences), anti-c-Rel 1:50 (sc-70, Santa Cruz), anti-HMGB1 1:100 (ab79823, Abcam). After rinsing, slides were incubated with the appropriate secondary antibodies for 1 hour at RT. Cells were washed in PBS and mounted in mounting medium Vectashield (Vector Laboratories). Negative control slides were incubated with blocking solution instead of the primary antibodies. Staining was visualized in a fluorescence microscope (Axiophot; Carl Zeiss, Jena, Germany). Quantification of fluorescence intensity was done with ImageJ Software (NIH, USA).

### Phagocytosis assay

Microglial cells (1 x 10^5^) were plated onto glass coverslips and treated with 100 nM NDP-MSH for 24 hours, then stimulated with either Pam_3_CSK_4_ or LPS (100 ng/ml) for 24 additional hours. 2 hours before ending the incubation period, 3 μl of a fluorescent carboxylated-latex beads suspension in complete DMEM were added to the cultures in a 100:1 bead to cell ratio. Cells were fixed in 4% PBS-PFA for 10 minutes at RT and processed for immunocytochemistry for the microglial marker CD11b. The number of beads per CD11b positive cell in each experimental group was counted in a Zeiss Axiophot fluorescence microscope under a 40X lens. Cells containing ten or more microspheres were considered positive for phagocytosis, as described elsewhere [24] and the proportion of phagocytosis-positive cells was calculated for each experimental group. To verify that the presence of beads within the cells was due to active phagocytosis, cells were incubated with the bead solution at 4°C for 2 hours, after which no latex beads were detected inside the cells (internal control not shown).

### Statistical analysis

Data were analysed by one sample t test, Student’s t test or one-way analysis of variance (ANOVA) followed by Bonferroni’s multiple comparisons test, as required by the experimental design. GraphPad Prism 5 Software was used (GraphPad Software, CA, USA). Differences with a value of *p*<0.05 were considered statistically significant.

### Ethics Statement

Experimental procedures were approved by the Institutional Committee for the Care and Use of Laboratory Animals (CICUAL) of the School of Medicine, University of Buenos Aires, Argentina (resolution n° 096/2010) and were carried out in compliance with the guidelines of the NIH Guide for the Care and Use of Laboratory Animals.

## Results

### Effect of NDP-MSH on the expression of M2 markers and inflammatory mediators on microglial cells

**Fig 1:**
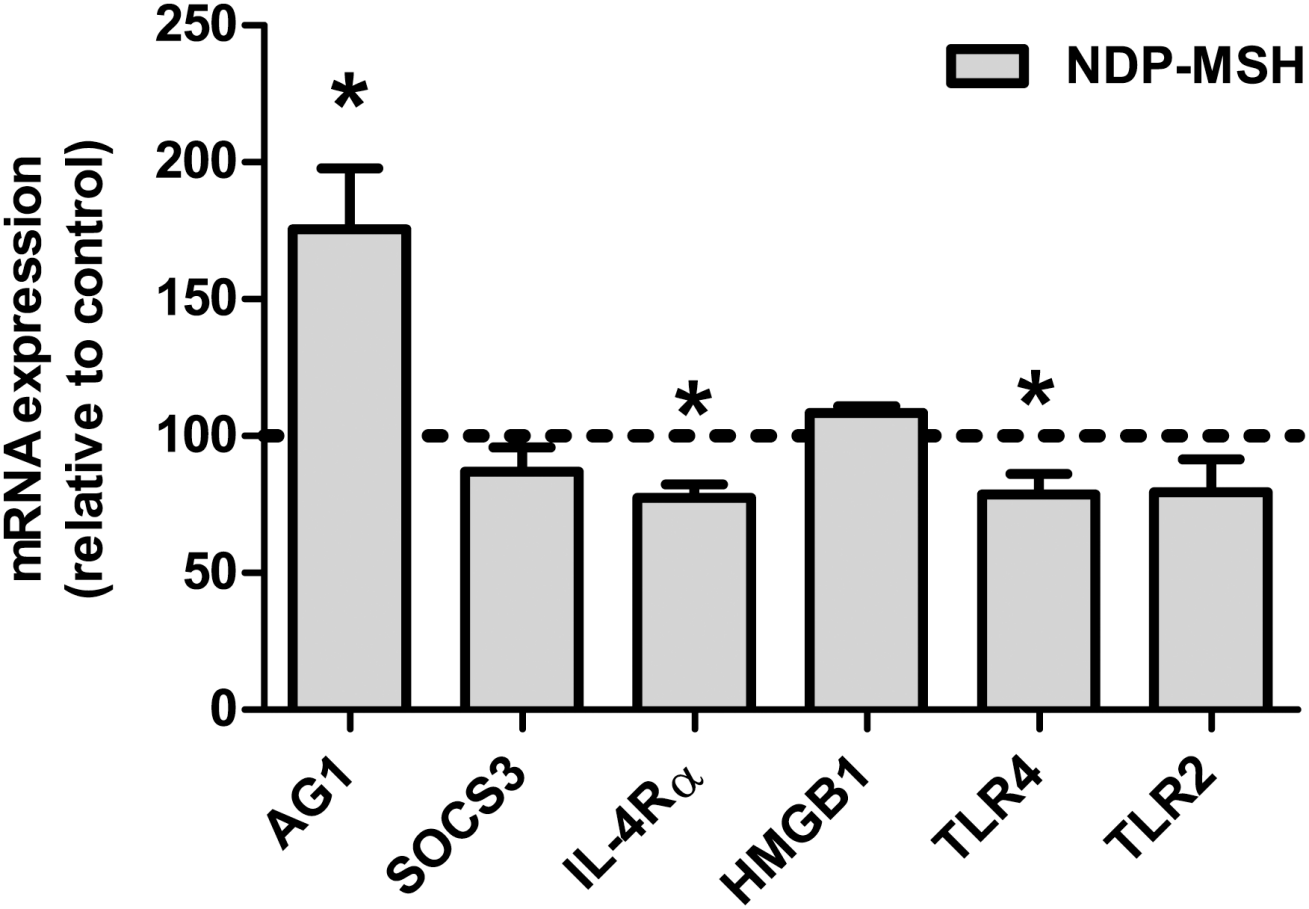
Effect of NDP-MSH on the expression of M2 markers and inflammatory mediators. Cells were treated with 100 nM NDP-MSH for 24 hours. Gene expression was assessed by RT-qPCR. Values are the mean ± SEM of at least 3 independent experiments and are expressed as the percentage of their respective controls (arbitrarily set at 100% and represented by the dotted line). Data were analysed by one-sample t test. *p<0.05 vs. control group.

To study the effect of NDP-MSH on microglial polarization we assessed the gene expression of the M2 markers *Ag1*, *Socs3* and *Il-4rα* and the inflammatory mediators *Hmgb1*, *Tlr2* and *Tlr4* by RT-qPCR. Treatment with NDP-MSH for 24 hours increased the expression of *Ag1* (Fig 1). Conversely, expression of *Tlr4* and *Il-4rα* was reduced by NDP-MSH (Fig 1). *Socs3*, *Tlr2* and *Hmgb1* expression levels were not modified by NDP-MSH (Fig 1). Induction of *Ag1* expression plus reduction in *Tlr4* expression, added to the previously described increase in PPAR-γ expression and IL-10 release [21], support a role for NDP-MSH in microglial polarization towards an alternative M2-like profile.

### NF-κB activation

**Fig 2:**
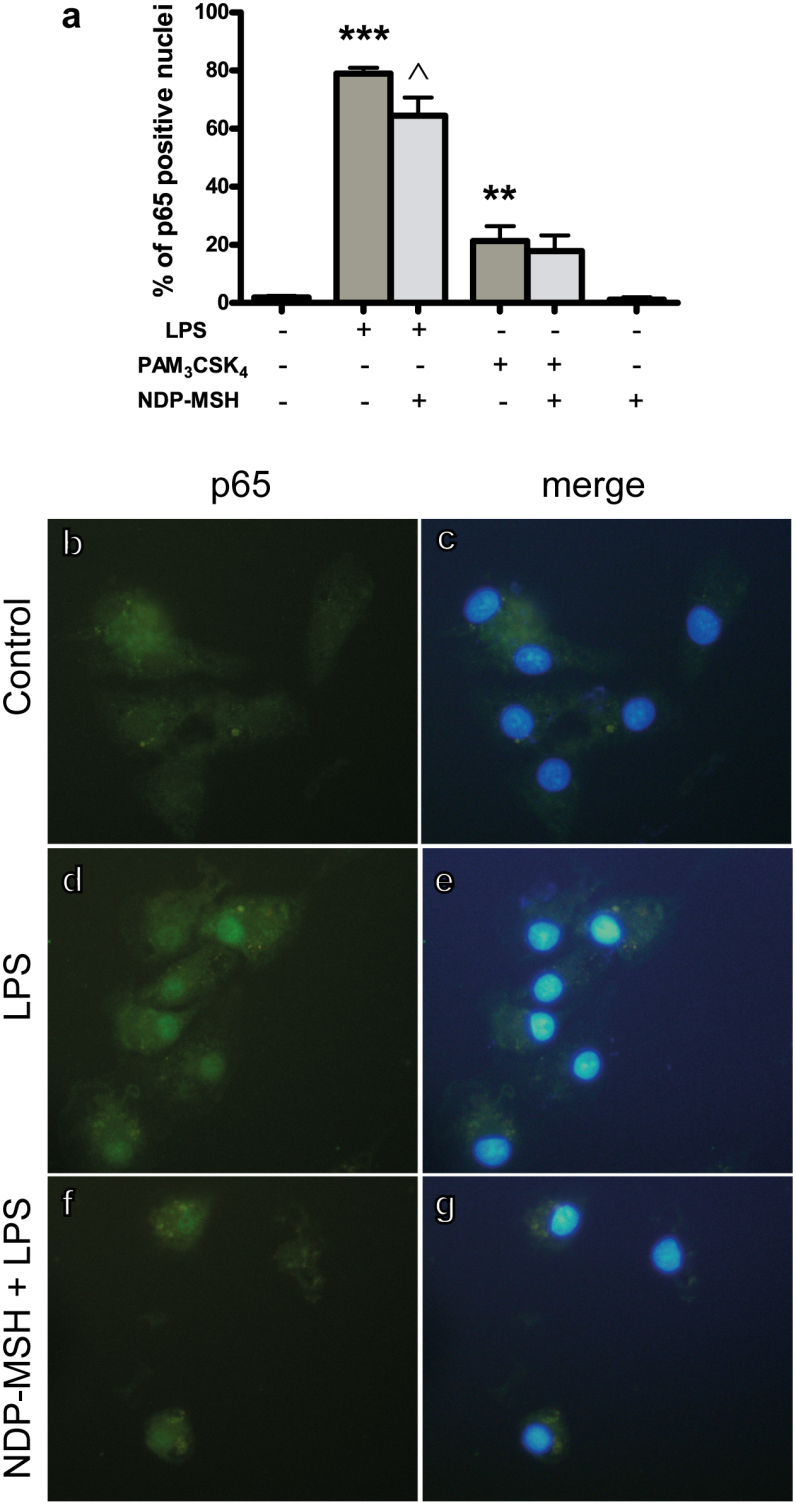
NDP-MSH prevents LPS-induced p65 nuclear translocation. Cells were treated for 24 hours with 100 nM NDP-MSH and then stimulated for 30 minutes with LPS (100 ng/ml) or Pam_3_CSK_4_ (100 ng/ml) and processed for p65 immunocytochemistry. **(a)** The percentage of p65-positive nuclei was calculated for each experimental group. Data are the mean ± SEM of 4 independent experiments and were analysed by one-way ANOVA followed by Bonferroni’s multiple comparisons test ***p<0.001, **p<0.01 vs. control; **^***p*<0.05 vs. LPS. Representative images are shown of the following groups: **(b)** and **(c)** Control; **(d)** and **(e)** LPS; **(f)** and **(g)** NDP-MSH + LPS. Green: p65. Blue: nuclei stained with DAPI.

We tested whether NDP-MSH could inhibit TLR-induced NF-κB activation by determining the intracellular localization of the p65 and c-Rel NF-κB subunits upon treatment with LPS or Pam_3_CSK_4_. Immunostaining for p65 was evenly distributed within the cytoplasm and absent from the nucleus in the untreated microglial cells (control group, Fig 2.b). Treatment with LPS strongly increased the percentage of p65-positive nuclei (Fig 2.a and 2.d) and pre-treatment with NDP-MSH reduced the percentage of p65-positive nuclei compared to LPS alone (Fig 2.a and 2.f). Treatment with Pam_3_CSK_4_ induced p65 nuclear translocation—although not as robustly as LPS—(Fig 2.a) and pre-treatment with NDP-MSH did not modify the percentage of p65-positive nuclei upon stimulation with Pam_3_CSK_4_ (Fig 2.a). NDP-MSH alone did not modify the percentage of p65-positive nuclei compared to the control group (Fig 2.a). These results indicate that NDP-MSH prevents TLR4-induced p65 activation.

**Fig 3:**
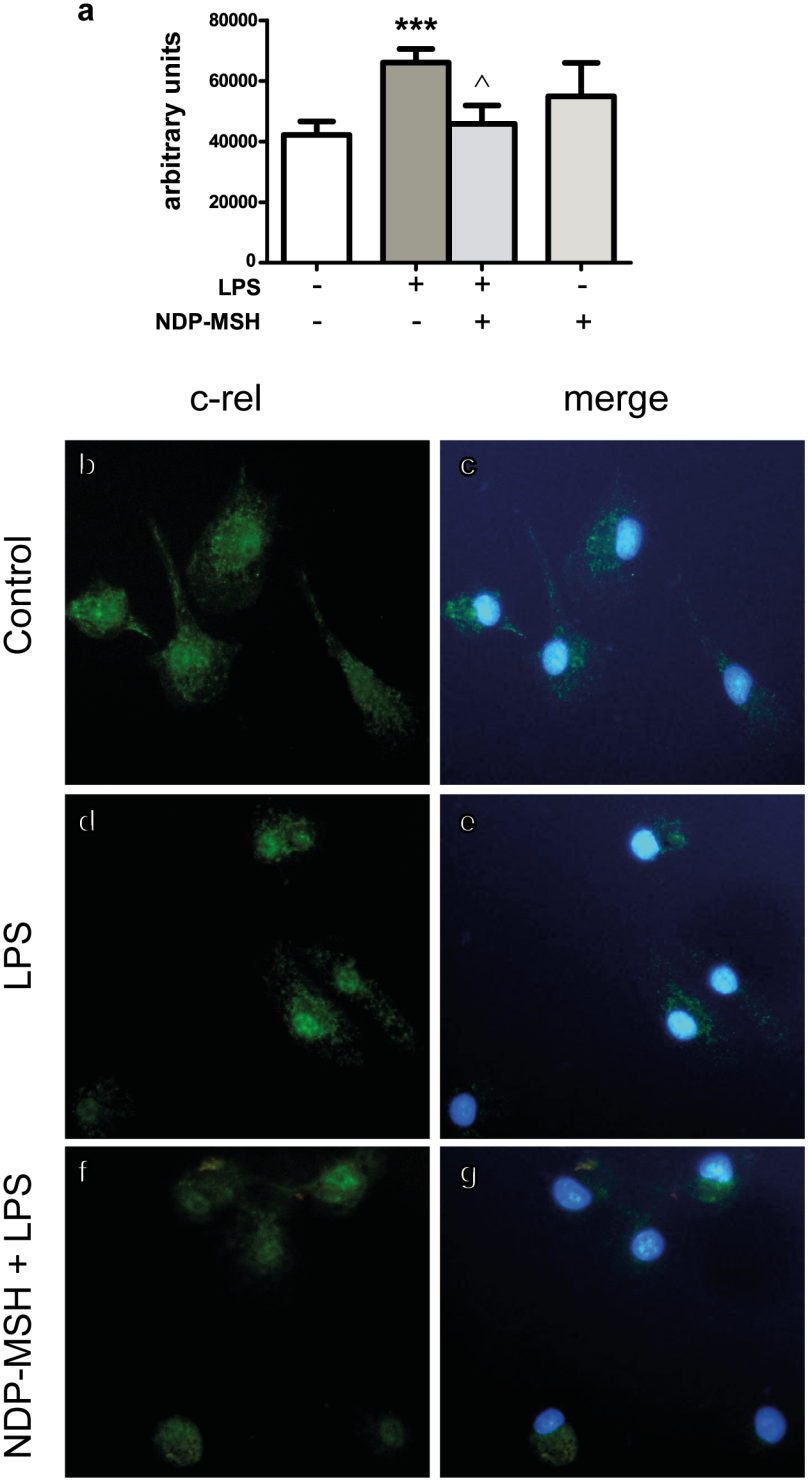
NDP-MSH prevents LPS-induced c-Rel nuclear translocation. Cells were treated for 24 hours with 100 nM NDP-MSH and then stimulated for 30 minutes with LPS (100 ng/ml) or Pam_3_CSK_4_ (100 ng/ml) and processed for c-Rel immunocytochemistry. **(a)** Nuclear fluorescence intensity was semi-quantified using ImageJ software. Data are the mean ± SEM of 4 independent experiments and were analysed by one-way ANOVA followed by Bonferroni’s multiple comparisons test. ***p<0.001 vs. control. ^*p*<0.01 vs. LPS. Representative images are shown of the following groups: **(b)** and **(c)** Control; **(d)** and **(e)** LPS; **(f)** and **(g)** NDP-MSH + LPS. Green: c-Rel. Blue: nuclei stained with DAPI.

Nuclear c-Rel immunostaining was evident in untreated microglial cells (Fig 3.b). Therefore, we decided to compare the intensity of nuclear c-Rel immunoreactivity rather than positive versus negative nuclei. Treatment with LPS increased nuclear c-Rel immunoreactivity (Fig 3.a and 3.d) and this effect was inhibited by NDP-MSH pre-treatment (Fig 3.a and 3.f), indicating that NDP-MSH also inhibits TLR4-induced c-Rel activation. Treatment with NDP-MSH alone had no significant effect on c-Rel immunoreactivity compared to the control group (Fig 3.a). On the other hand, treatment with Pam_3_CSK_4_ did not increase c-Rel nuclear immunoreactivity compared to untreated cells (data not shown).

### Cytokine release

**Fig 4:**
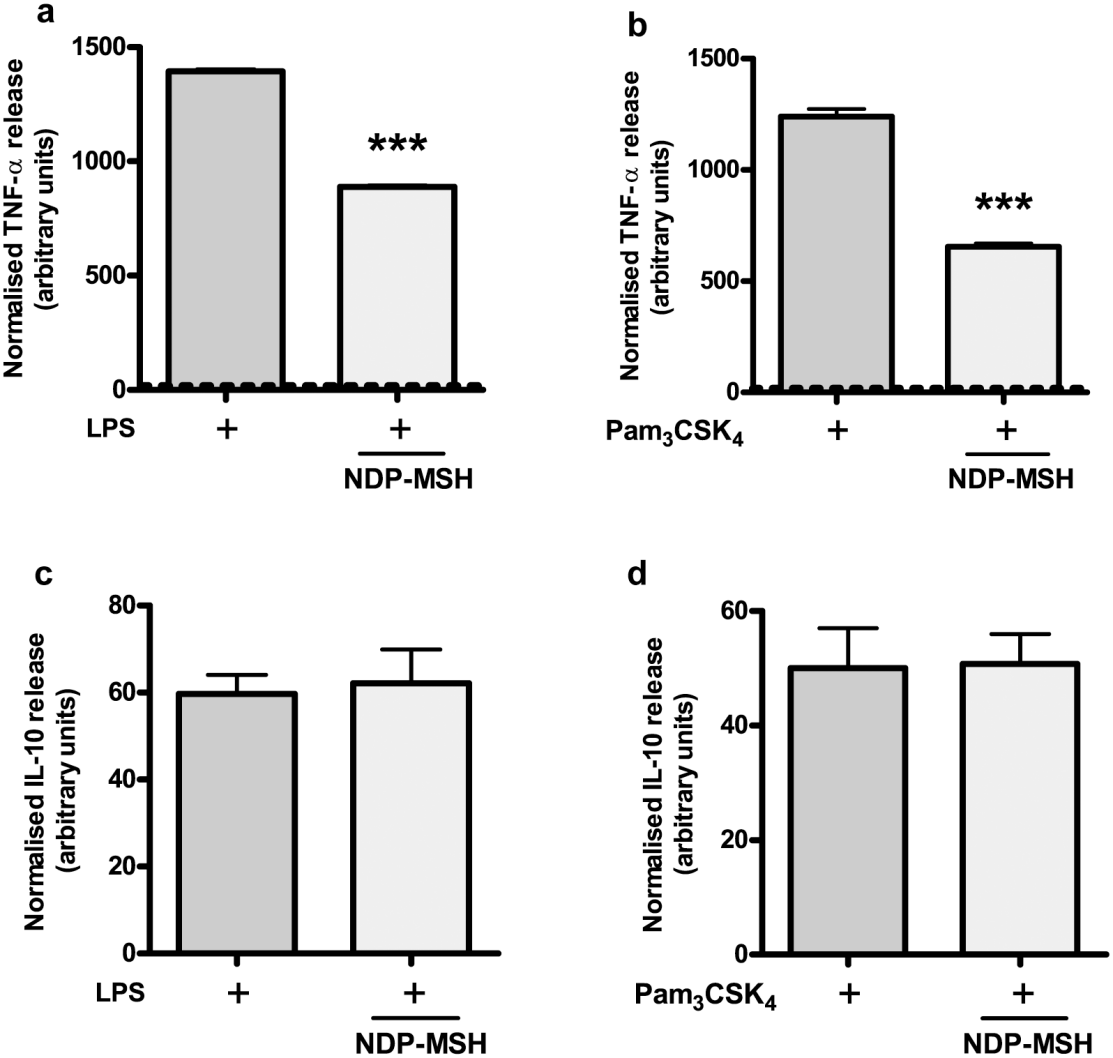
NDP-MSH inhibits TLR4- and TLR2-induced TNF-α release but not IL-10 release. Cells were pre-treated for 24 hours with 100 nM NDP-MSH and then stimulated with LPS (100 ng/ml) or Pam_3_CSK_4_ (100 ng/ml). TNF-α release into the culture supernatant after 4.5 hours **(a)** and **(b)** and IL-10 release after 24 hours **(c)** and **(d)** were assessed by ELISA and normalised to the viability values obtained by the MTT assay for each experimental group (MTT values not shown). The dotted line in **(a)** and **(b)** represents values of TNF-α release for control groups. Data are the mean ± SEM of 6 replicates and were analysed by Student’s t test. Experiments were performed twice. **(a)** ***p<0.001 vs. LPS. **(b)** ***p<0.001 vs. Pam_3_CSK_4_.

In order to determine the effect of NDP-MSH on TLR-induced cytokine release, we determined the concentration of the proinflammatory cytokine (and M1/M2b marker) TNF-α in the culture supernatant upon TLR-stimulation. We also assessed the release of the anti-inflammatory mediator IL-10 and of the M2-inducer IL-4 as a possible autocrine mechanism of NDP-MSH-induced alternative polarization. Both LPS and Pam_3_CSK_4_ induced the release of TNF-α to the culture supernatant compared to the control group, and pre-treatment with NDP-MSH inhibited TLR4- and TLR2-induced TNF-α release (Fig 4.a and 4.b, respectively). On the other hand, NDP-MSH did not modify TLR4 and TLR2-induced IL-10 release (Fig 4.c and 4.d, respectively), suggesting that the melanocortin is selectively inhibiting the release of a proinflammatory mediator rather than globally decreasing microglial activity. IL-10 release in the control groups was below the detection limit of the commercial kit (15.6 pg/ml) and therefore these groups are not shown. On the other hand, we detected no IL-4 release either in the control group, or in the NDP-MSH-, LPS- or Pam_3_CSK_4_-treated cells, and NDP-MSH also failed to induce IL-4 release in mixed glial cell cultures (data not shown). This result indicates that cultured rat microglial cells do not release IL-4 in the conditions tested and suggests that IL-4 is not a mediator of NDP-MSH effects *in vitro*.

### HMGB1 intracellular localization

**Fig 5:**
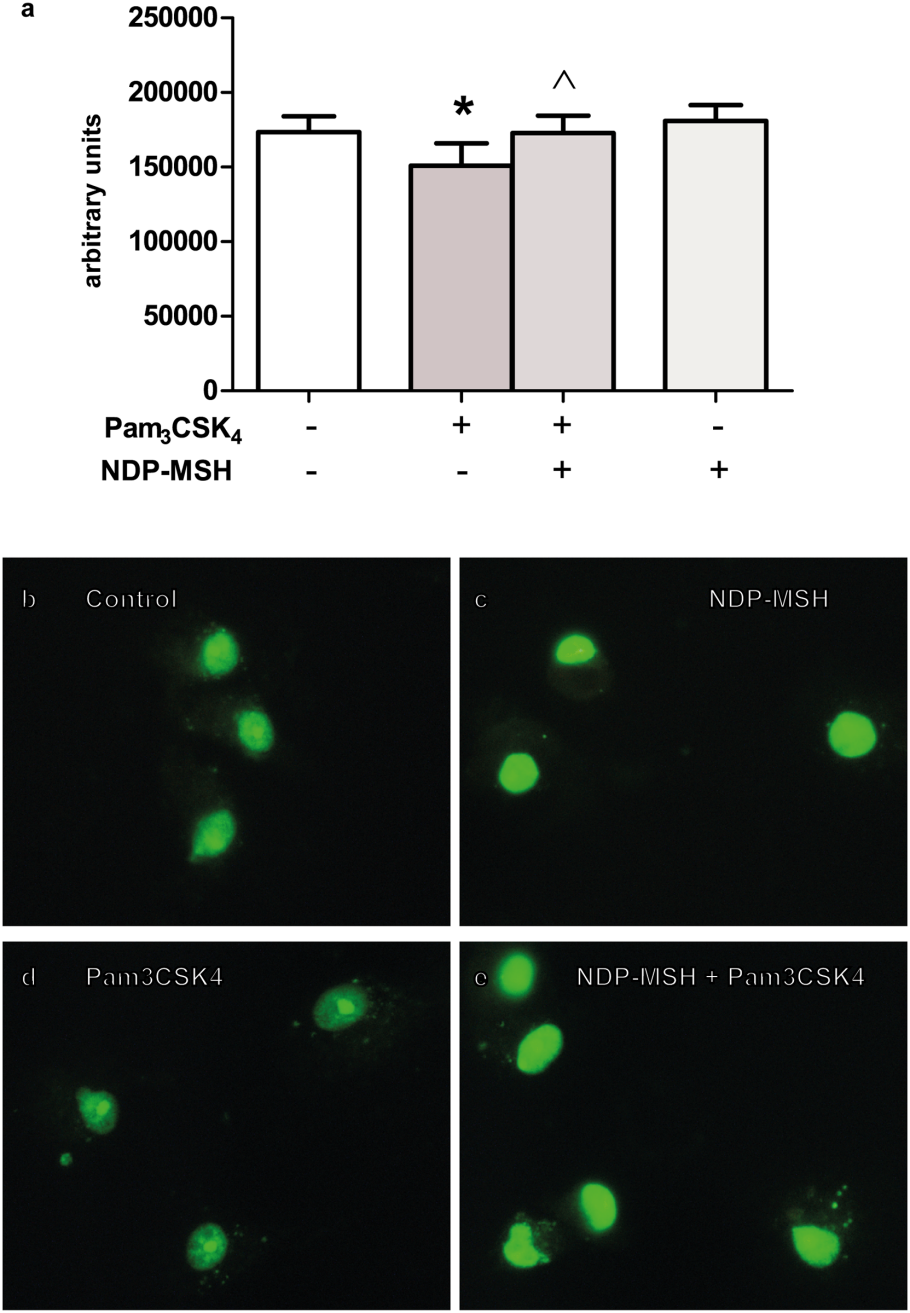
NDP-MSH prevents Pam_3_CSK_4_-induced HMGB1 nucleus-to cytoplasm translocation. Cells were treated for 24 hours with 100 nM NDP-MSH and then stimulated for another 24 hours with LPS (100 ng/ml) or Pam_3_CSK_4_ (100 ng/ml). Cells were immunostained for HMGB1 and nuclear fluorescence intensity was semi-quantified using ImageJ software. **(a)** Data are the mean ± SEM of 5 independent experiments and were analysed by one-way ANOVA followed by Bonferroni’s multiple comparisons test. *p<0.05 vs. control, ^*p*<0.05 vs. Pam_3_CSK_4_. Representative images are shown of the following groups: **(b)** Control; **(c)** NDP-MSH; **(d)** Pam_3_CSK_4;_ **(e)** NDP-MSH + Pam_3_CSK_4_. Green: HMGB1.

HMGB1 is a nuclear protein that can be released to the extracellular milieu upon stimulation by TLR agonists and act as a proinflammatory cytokine [25]. Since HMGB1 must translocate from nucleus to cytoplasm prior to its release, the effect of NDP-MSH on TLR-induced HMGB1 translocation was investigated. Cells were pre-treated with or without NDP-MSH for 24 hours and then stimulated with LPS or Pam_3_CSK_4_ for 24 additional hours. HMGB1 immunoreactivity was observed in the nuclei of all cells in the control group, and some cells also displayed a faint punctuate pattern of HMGB1 in the cytoplasm (Fig 5.a and 5.b). Upon stimulation with Pam_3_CSK_4_ we observed a decrease in nuclear HMGB1 fluorescence intensity and an increase in the punctuate HMGB1 pattern in the cytoplasmic region (Fig 5.a and 5.d) suggesting an active release of HMGB1 from nucleus to cytoplasm. In the NDP-MSH-pre-treated group there was an increase in HMGB1 nuclear fluorescence intensity to the levels of the control group, suggesting that NDP-MSH prevented Pam_3_CSK_4_-induced HMGB1 release from nucleus to cytoplasm (Fig 5.a and 5.e). NDP-MSH alone had no effect on HMGB1 intracellular localization compared to the control group (Fig 5.a and 5.c). These results suggest that NDP-MSH inhibits nuclear release of HMGB1 upon TLR2 stimulation. LPS did not significantly induce HMGB1 translocation in the conditions tested (data not shown).

### Microglial phagocytosis

**Fig 6:**
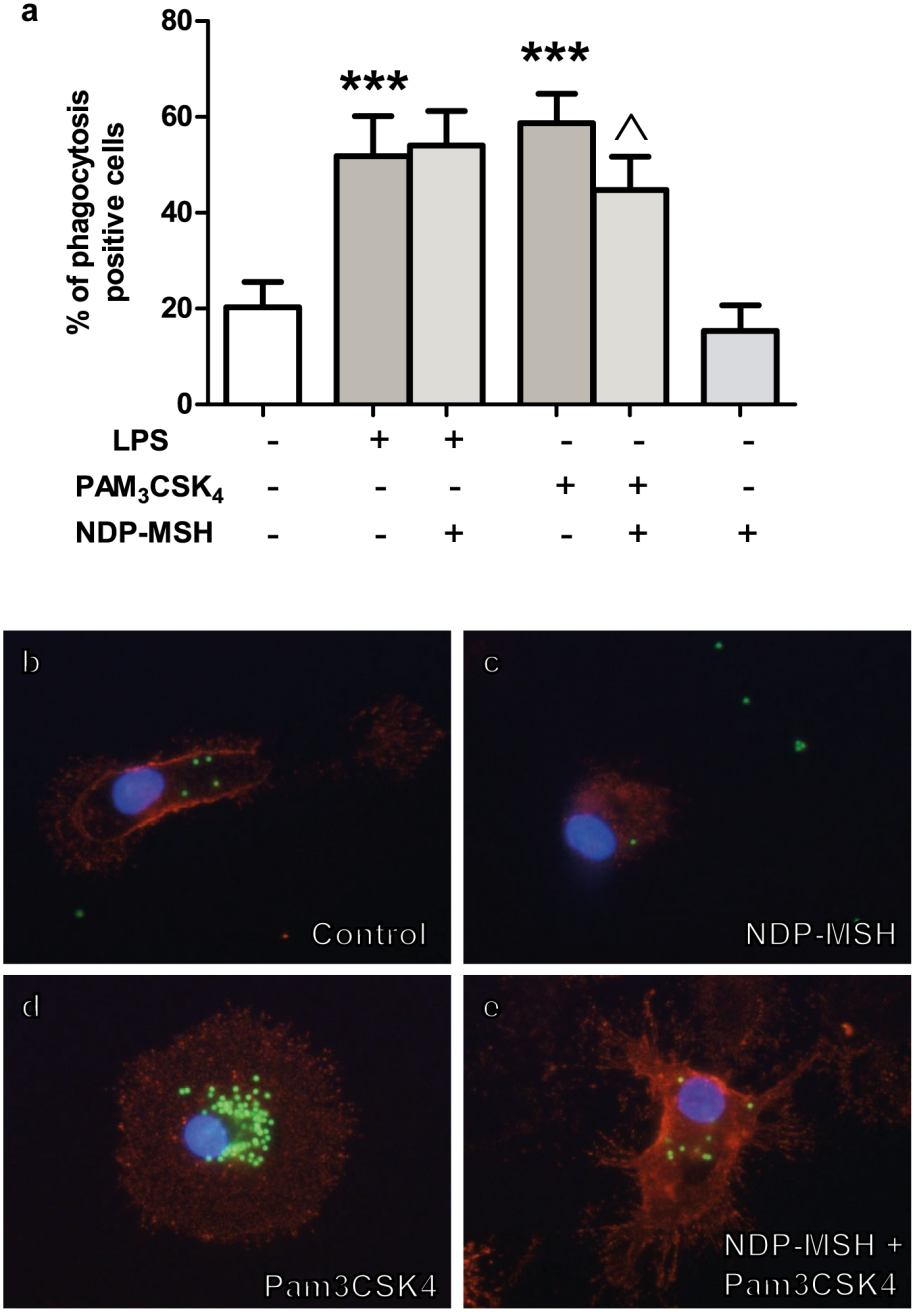
NDP-MSH decreases Pam_3_CSK_4_-induced microglial phagocytosis of latex beads. Cells were treated for 24 hours with 100 nM NDP-MSH and then stimulated for another 24 hours with LPS (100 ng/ml) or Pam_3_CSK_4_ (100 ng/ml). The percentage of phagocytosis-positive cells was calculated by counting the number of CD11b^+^ cells containing ten or more microspheres as described in Materials and Methods. At least 10 fields were counted for each experimental group. **(a)** Values are the mean ± SEM of 8 independent experiments. Data were analysed by one-way ANOVA followed by Bonferroni’s multiple comparisons test. ***p<0.001 vs. control group, ^*p*<0.05 vs. Pam_3_CSK_4_. Representative images are shown of the following groups: **(b)** Control; **(c)** NDP-MSH; **(d)** Pam_3_CSK_4_; **(e)** NDP-MSH + Pam_3_CSK_4_. Red: Cd11b. Green: FITC-conjugated microspheres. Blue: nuclei stained with DAPI.

Both Pam_3_CSK_4_ and LPS are known stimulators of microglial phagocytic activity [26, 27]. The effect of NDP-MSH on TLR-induced phagocytosis was investigated through the uptake of fluorescent microspheres; cells containing 10 or more microspheres were considered positive for phagocytosis, as described in Materials and Methods. As expected, the percentage of cells positive for phagocytosis significantly increased upon stimulation with both LPS (Fig 6.a) and Pam_3_CSK_4_ (Fig 6.a and 6.d). The effect of Pam_3_CSK_4_ was inhibited by pre-treatment with NDP-MSH (Fig 6.a and 6.e). However, NDP-MSH did not affect LPS-induced phagocytosis (Fig 6.a), suggesting that the pathways by which the two TLR agonists initiate microsphere uptake involve different mediators. Finally, NDP-MSH alone had no effect on microglial phagocytosis (Fig 6.a and 6.c).

## Discussion

Modulation of microglial activation is of major relevance in the context of inflammatory and neurodegenerative disorders in the CNS, such as AD, multiple sclerosis (MS) and ischemic damage, amongst others. **In the present study we show that the synthetic α-MSH analog, NDP-MSH, inhibits some TLR2- and TLR4-triggered inflammatory mechanisms in cultured rat microglial cells and promotes development of an alternative M2-like phenotype**. Targeting central MCRs present in neurons and glial cells constitutes a new approach to treating neuroinflammation. The effectiveness of selective MC4R agonists in modulating inflammatory processes plus their low toxicity strongly positions these molecules as useful instruments for treatment of CNS disorders with an inflammatory component [12].

Classically, microglial cells were classified based on morphological features often associated with distinct functional roles [28]. However, in recent years great efforts have been made to identify more specific features to help describe the extremely diverse phenotypes that microglial cells and macrophages can acquire in response to a particular set of stimuli. As a consequence, two categories have been proposed in order to describe the two main states in which these cells can be found, termed M1 and M2. The M1 category describes a population of macrophages or microglial cells that become *classically* activated in response to T_H_1 cytokines such as IFN-γ, bacterial moieties such as LPS or other TLR agonists, and whose main feature is production of proinflammatory mediators such as IL-1β, TNF-α and IL-12 [29]. The M2 category comprises a population of cells whose main functions include immunomodulation, immunosuppression and tissue repair [30]. It is in turn divided into four subcategories: M2a, M2b, M2c and M2d each possessing its own set of markers and putative predominant functions (for a comprehensive review see: [1]). The M2a profile is driven mainly by T_H_2 cytokines such as IL-13 and IL-4. The M2b phenotype is induced by LPS and immune complexes and the M2c by IL-10 and TGF-β, whereas the M2d is applied to tumour associated macrophages which typically display M2-like properties [31]. However, increasing evidence indicates that microglial cells are a highly plastic and dynamic population and that fitting their behaviour into one specific category is a difficult task, a fact highlighted by the great overlapping of markers between the different proposed phenotypes.

We previously showed that microglial cells express the MC4 receptor and that they respond to the melanocortin analog NDP-MSH by releasing the anti-inflammatory cytokine IL-10 and by increasing the expression of the nuclear receptor PPAR-γ [21]. Both IL-10 and PPAR-γ are—together with AG1—markers of the M2a/c alternative profiles in microglia and macrophages [1]. Here we show that upon NDP-MSH stimulation microglial cells increase the expression of *Ag1*, further supporting a polarizing role of melanocortins towards a M2-like phenotype. Similarly, it has been shown that α-MSH and neuropeptide Y derived from the ocular microenvironment alternatively activate resting macrophages to co-express AG1 and NOS2 [32]. Activity of AG1 leads to generation of L-ornithine and polyamines, important mediators of collagen production for tissue repair and in cell differentiation and proliferation, respectively [33], thus influencing key steps of the inflammatory response. On the other hand, NDP-MSH reduced expression of *Il-4rα* and did not modify that of *Socs3*, both markers that have been associated with the M2b/c phenotypes [29]. NDP-MSH also reduced expression of *Tlr4* in microglial cells, although it did not significantly affect expression of *Tlr2*. TLR4 has been reported to mediate microglial activation and associated neuroinflammation [34]. Also, microglial TLR4 is required for efficient lymphocyte recruitment to the CNS in response to LPS [35]. Thus, the finding that the MC4R agonist can decrease microglial TLR4 expression suggests that melanocortins could be useful for reducing TLR4-mediated neurotoxicity in neurodegenerative diseases. Previous studies revealed an important role for HMGB1 in promoting M1 macrophage polarization [36]. In our system, no changes in *Hmgb1* expression levels were found after treatment with NDP-MSH. Collectively, our data indicate that NDP-MSH polarizes microglial cells towards an alternative profile which shares features with the M2a and M2c subcategories. However, we must note that changes in mRNA expression do not necessarily reflect changes in protein expression; hence further investigation is required for better understanding the effect of NDP-MSH on protein expression of the different microglial phenotype markers.

Stimulation of TLR2 and TLR4 leads to activation of the transcription factor NF-κB. NF-κB family is composed of five subunits, p65/RelA, p50, p52, c-Rel and RelB. Upon activation, these subunits can form homo or heterodimers and translocate to the nucleus where they modulate expression of several genes involved in cell survival and in the inflammatory response [37]. In the present work we found that NDP-MSH prevented LPS-induced p65 nuclear translocation. It has been reported that α-MSH inhibits NF-κB activation in several culture systems, including monocytes [38], macrophages, neutrophils and Jurkat cells [39]. However, α-MSH was also reported to fail to prevent NF-κB activation in a glioma cell line, where its inhibitory effects were instead explained by a reduction in the LPS co-receptor CD14 [39]. In addition, previous results from our group demonstrated that α-MSH did not prevent the increase in nuclear p65 protein levels induced by LPS+IFN-γ in primary cultured rat astrocytes [22], further suggesting that this protective mechanism might be cell-specific.

Our results also show that LPS stimulation increased c-Rel immunoreactivity within the nucleus and that pre-treatment with NDP-MSH prevented this effect. c-Rel is essential for production of the IL-12p40 subunit in LPS-treated microglial cells [40] and in macrophages [41]. This subunit is shared by proinflammatory cytokines IL-12 and IL-23. IL-12 stimulates T_H_1 T-cell development and thereby helps to establish a nexus between innate and adaptive immunity [42]. IL-23 promotes expansion of the T_H_17 T-cell population, which is linked to autoimmune inflammatory disease such as MS [43]. In experimental autoimmune encephalomyelitis (EAE), an animal model of MS, treatment with α-MSH diminishes the severity of the disease [44]. Thus, specific inhibition of microglial c-Rel by NDP-MSH in an inflammatory context may prevent amplification of the immune response and could be linked to beneficial effects of melanocortins in CNS autoimmune inflammatory disease.

In our system, the effects of NDP-MSH on LPS-induced p65 and c-Rel translocation may be partially explained by the reduction in *Tlr4* expression. In addition, IL-10 has been shown to inhibit LPS-induced p65 (and p50) activation in microglial cells [45]. Since we previously showed that NDP-MSH induces IL-10 release from primary cultured microglial cells [21], it is possible that this cytokine may be partially responsible for the effect of NDP-MSH on TLR4-induced p65 translocation. Our findings raise the possibility that a reduction in microglial TLR4 expression and of p65 and c-Rel activation may be linked to the neuroprotective effects of melanocortins in acute and chronic inflammation in the brain.

Nuclear translocation of p65 in response to stimulation with Pam_3_CSK_4_ was significantly increased, although not as robustly as with LPS stimulation. However, the effect of Pam_3_CSK_4_ was not prevented by NDP-MSH. This lack of effect of NDP-MSH is consistent with the absence of an inhibitory action on *Tlr2* expression and may also indicate that different mechanisms are involved in the induction of p65 translocation upon TLR2 and TLR4 ligation.

Pam_3_CSK_4_ has been demonstrated to strongly induce c-Rel nuclear translocation in macrophages after 2 hours of incubation [46]. However, treatment with Pam_3_CSK_4_ did not significantly increase c-Rel immunoreactivity within the nucleus in our experimental conditions. The discrepancies between previous results and ours could be due to the different cell type involved.

TLR-induced activation of NF-κB typically results in a rapid release of the proinflammatory cytokine TNF-α, followed by a delayed production of the immunomodulatory factor IL-10, which acts as a negative modulator of the inflammatory response [47]. Our results show that pre-treatment with NDP-MSH inhibits early TLR2- and TLR4-induced TNF-α release, whereas it does not affect late IL-10 release. NF-κB subunit p65 has been proposed to mediate Pam_3_CSK_4_- and LPS-induced TNF-α production in microglial cells [48] and macrophages [10], respectively. In contrast, IL-10 production upon Pam_3_CSK_4_ stimulation has been linked to activation of the non canonical (p100/p52) pathway in monocytes [49] and of the p50/p50 homodimers in macrophages [50]. The inhibitory effect of NDP-MSH on LPS-induced TNF-α release could therefore be explained by its effect on p65 activation. However, even though NDP-MSH did not prevent Pam_3_CSK_4_-induced p65 nuclear translocation, it still decreased Pam_3_CSK_4_-induced TNF-α release. One possible explanation for this effect is that NDP-MSH may be inhibiting NF-κB transcriptional activity without inhibiting its translocation to the nucleus, a mechanism previously described for IL-10 in macrophages [51]. Another possibility is that NDP-MSH may also be affecting translocation or activation of the NF-κB p50 subunit, a molecule suggested to partner with p65 in the induction of TNF-α transcription in monocytes [52, 53].

CNS-derived IL-4 is a known inducer of the alternative M2 phenotype in microglial cells [54]. Working with the hypothesis that NDP-MSH drives microglial cells towards an alternative activation profile and knowing that microglial cells express the IL-4 receptor [55], we set out to determine whether IL-4 could be mediating the effects of NDP-MSH on microglial cells in an autocrine manner. However, we detected no IL-4 release to the culture supernatant either in the control group or in the NDP-MSH-, LPS- or Pam_3_CSK_4_-treated cells. Furthermore, NDP-MSH also failed to induce IL-4 release in mixed glial cell cultures (data not shown). Thus, we conclude that cultured rat microglial cells do not release IL-4 in the conditions tested and that the effects of NDP-MSH observed are not mediated by this cytokine. In animal models of inflammatory brain disease such as EAE, microglial cells have been proved to be an important source of IL-4 [54], suggesting that factors present in the CNS microenvironment (absent in the culture system) are essential for production of this cytokine.

HMGB1 is a highly conserved nuclear protein belonging to a group of molecules termed alarmins. Within the nucleus it acts as a regulator of DNA structure and transcription [56]. HMGB1 can be released to the extracellular milieu during traumatic cell death or upon stimulation by a number of inflammatory mediators such as TLR agonists [25]. Once outside the cell, HMGB1 can form complexes with other proinflammatory mediators such as IL-1β or LPS [57] and potentiate TLR-mediated proinflammatory effects, promote tissue damage and immune reactions [5, 25] and, as mentioned before, facilitate M1 macrophage polarization [36]. In view of these effects, reducing the amount of HMGB1 released upon TLR stimulation could be beneficial in the context of neuroinflammation. Our results showed that treatment with Pam_3_CSK_4_ induced HMGB1 nucleus-to-cytoplasm translocation (a phenomenon occurring prior to its release) and we observed that NDP-MSH pre-treatment inhibited this effect. This result suggests a protective role of the melanocortin by potentially hampering HMGB1 release, thereby preventing the delayed potentiation of the TLR-triggered inflammatory response, a possibility that requires further investigation.

Microglial activation through TLR2 and TLR4 increases phagocytosis [26, 27]. Here we show that treatment with NDP-MSH prior to stimulation with Pam_3_CSK_4_ resulted in reduced phagocytic activity compared to non-pre-treated cells. Phagocytosis in an inflammatory context has generally been considered a beneficial process due to removal of dying cells and cellular debris which ultimately facilitates the return to homeostasis. Furthermore, certain chronic neurodegenerative diseases appear to be associated with dysfunctional microglial phagocytosis [58]. However, recent evidence suggests that microglial phagocytosis in an inflammatory context may not always be desirable and may actually cause neuronal death by a mechanism termed *phagoptosis* [59]. In this study, the authors demonstrated that microglial activation by TLR2 and TLR4 agonists stimulates neurons to reversibly expose phosphatidyl-serine which in turn acts as an “eat me” signal for activated microglial cells and results in phagocytosis of the otherwise healthy neurons [59]. This finding suggests that prevention of TLR-induced microglial activation could also prevent neuronal death by phagoptosis in an inflammatory context. In view of these results, modulation of microglial activation by NDP-MSH could prove beneficial in neuroinflammatory disorders linked to neuron loss by phagocytosis such as brain ischemia [60]. On the other hand, NDP-MSH did not inhibit LPS-induced phagocytosis, suggesting that it is acting on a TLR2-triggered mechanism most likely not involved in TLR4-mediated phagocytosis.

To summarize, our data show that NDP-MSH prevented several proinflammatory mechanisms triggered by TLR2 and TLR4 activation and promoted development of an alternative M2-like phenotype on rat microglial cells. Our findings provide new insights into mechanisms through which melanocortins modulate microglial activation upon TLR stimulation and support microglial MCRs as potential targets in the treatment of neuroinflammatory disorders.

## References

[1] Franco R, Fernandez-Suarez D. Alternatively activated microglia and macrophages in the central nervous system. Prog Neurobiol. 2015;131:65–86. 10.1016/j.pneurobio.2015.05.003 . 26067058

[2] Olson JK, Miller SD. Microglia initiate central nervous system innate and adaptive immune responses through multiple TLRs. J Immunol. 2004;173(6):3916–24. Epub 2004/09/10. 173/6/3916 [pii]. . 15356140 10.4049/jimmunol.173.6.3916

[3] Kumar H, Kawai T, Akira S. Pathogen recognition by the innate immune system. Int Rev Immunol. 2011;30(1):16–34. Epub 2011/01/18. 10.3109/08830185.2010.529976 . 21235323

[4] Bianchi ME. DAMPs, PAMPs and alarmins: all we need to know about danger. J Leukoc Biol. 2007;81(1):1–5. 10.1189/jlb.0306164 . 17032697

[5] Park JS, Svetkauskaite D, He Q, Kim JY, Strassheim D, Ishizaka A, et al Involvement of toll-like receptors 2 and 4 in cellular activation by high mobility group box 1 protein. J Biol Chem. 2004;279(9):7370–7. Epub 2003/12/09. 10.1074/jbc.M306793200 M306793200 [pii]. . 14660645

[6] Lehnardt S. Innate immunity and neuroinflammation in the CNS: the role of microglia in Toll-like receptor-mediated neuronal injury. Glia. 2010;58(3):253–63. 10.1002/glia.20928 . 19705460

[7] Gurley C, Nichols J, Liu S, Phulwani NK, Esen N, Kielian T. Microglia and Astrocyte Activation by Toll-Like Receptor Ligands: Modulation by PPAR-gamma Agonists. PPAR Res. 2008;2008:453120 Epub 2008/06/28. 10.1155/2008/453120 18584038 PMC2435222

[8] Hayden MS, Ghosh S. Shared principles in NF-kappaB signaling. Cell. 2008;132(3):344–62. 10.1016/j.cell.2008.01.020 . 18267068

[9] Liou HC, Hsia CY. Distinctions between c-Rel and other NF-kappaB proteins in immunity and disease. BioEssays: news and reviews in molecular, cellular and developmental biology. 2003;25(8):767–80. 10.1002/bies.10306 . 12879447

[10] Collart MA, Baeuerle P, Vassalli P. Regulation of tumor necrosis factor alpha transcription in macrophages: involvement of four kappa B-like motifs and of constitutive and inducible forms of NF-kappa B. Molecular and cellular biology. 1990;10(4):1498–506. 2181276 10.1128/mcb.10.4.1498-1506.1990 PMC362253

[11] Couper KN, Blount DG, Riley EM. IL-10: the master regulator of immunity to infection. J Immunol. 2008;180(9):5771–7. Epub 2008/04/22. 180/9/5771 [pii]. . 18424693 10.4049/jimmunol.180.9.5771

[12] Lasaga M, Debeljuk L, Durand D, Scimonelli TN, Caruso C. Role of alpha-melanocyte stimulating hormone and melanocortin 4 receptor in brain inflammation. Peptides. 2008;29(10):1825–35. Epub 2008/07/16. S0196-9781(08)00269-6 [pii] 10.1016/j.peptides.2008.06.009 . 18625277

[13] Giuliani D, Leone S, Mioni C, Bazzani C, Zaffe D, Botticelli AR, et al Broad therapeutic treatment window of [Nle(4), D-Phe(7)]alpha-melanocyte-stimulating hormone for long-lasting protection against ischemic stroke, in Mongolian gerbils. Eur J Pharmacol. 2006;538(1–3):48–56. Epub 2006/05/02. S0014-2999(06)00294-9 [pii] 10.1016/j.ejphar.2006.03.038 . 16647700

[14] Giuliani D, Ottani A, Mioni C, Bazzani C, Galantucci M, Minutoli L, et al Neuroprotection in focal cerebral ischemia owing to delayed treatment with melanocortins. Eur J Pharmacol. 2007;570(1–3):57–65. Epub 2007/06/26. S0014-2999(07)00626-7 [pii] 10.1016/j.ejphar.2007.05.025 . 17588564

[15] Caruso C, Mohn C, Karara AL, Rettori V, Watanobe H, Schioth HB, et al Alpha-melanocyte-stimulating hormone through melanocortin-4 receptor inhibits nitric oxide synthase and cyclooxygenase expression in the hypothalamus of male rats. Neuroendocrinology. 2004;79(5):278–86. Epub 2004/06/26. 10.1159/000079321 79321 [pii]. . 15218320

[16] Giuliani D, Bitto A, Galantucci M, Zaffe D, Ottani A, Irrera N, et al Melanocortins protect against progression of Alzheimer's disease in triple-transgenic mice by targeting multiple pathophysiological pathways. Neurobiol Aging. 2014;35(3):537–47. Epub 2013/10/08. 10.1016/j.neurobiolaging.2013.08.030 S0197-4580(13)00375-8 [pii]. . 24094579

[17] Gonzalez PV, Schioth HB, Lasaga M, Scimonelli TN. Memory impairment induced by IL-1beta is reversed by alpha-MSH through central melanocortin-4 receptors. Brain Behav Immun. 2009;23(6):817–22. Epub 2009/03/12. S0889-1591(09)00069-5 [pii] 10.1016/j.bbi.2009.03.001 . 19275930

[18] Gonzalez P, Machado I, Vilcaes A, Caruso C, Roth GA, Schioth H, et al Molecular mechanisms involved in interleukin 1-beta (IL-1beta)-induced memory impairment. Modulation by alpha-melanocyte-stimulating hormone (alpha-MSH). Brain Behav Immun. 2013;34:141–50. Epub 2013/08/24. 10.1016/j.bbi.2013.08.007 S0889-1591(13)00418-2 [pii]. . 23968970

[19] Machado I, Gonzalez PV, Vilcaes A, Carniglia L, Schioth HB, Lasaga M, et al Interleukin-1beta-induced memory reconsolidation impairment is mediated by a reduction in glutamate release and zif268 expression and alpha-melanocyte-stimulating hormone prevented these effects. Brain Behav Immun. 2015;46:137–46. 10.1016/j.bbi.2015.01.012 . 25637483

[20] Delgado R, Carlin A, Airaghi L, Demitri MT, Meda L, Galimberti D, et al Melanocortin peptides inhibit production of proinflammatory cytokines and nitric oxide by activated microglia. J Leukoc Biol. 1998;63(6):740–5. Epub 1998/06/10. . 9620667 10.1002/jlb.63.6.740

[21] Carniglia L, Durand D, Caruso C, Lasaga M. Effect of NDP-alpha-MSH on PPAR-gamma and -beta expression and anti-inflammatory cytokine release in rat astrocytes and microglia. PLoS One. 2013;8(2):e57313 Epub 2013/03/08. 10.1371/journal.pone.0057313 PONE-D-12-28242 [pii]. 23468969 PMC3582497

[22] Caruso C, Carniglia L, Durand D, Gonzalez PV, Scimonelli TN, Lasaga M. Melanocortin 4 receptor activation induces brain-derived neurotrophic factor expression in rat astrocytes through cyclic AMP-protein kinase A pathway. Mol Cell Endocrinol. 2012;348(1):47–54. Epub 2011/08/02. S0303-7207(11)00435-7 [pii] 10.1016/j.mce.2011.07.036 . 21803120

[23] Livak KJ, Schmittgen TD. Analysis of relative gene expression data using real-time quantitative PCR and the 2(-Delta Delta C(T)) Method. Methods. 2001;25(4):402–8. Epub 2002/02/16. 10.1006/meth.2001.1262 S1046-2023(01)91262-9 [pii]. . 11846609

[24] Karlstetter M, Nothdurfter C, Aslanidis A, Moeller K, Horn F, Scholz R, et al Translocator protein (18 kDa) (TSPO) is expressed in reactive retinal microglia and modulates microglial inflammation and phagocytosis. J Neuroinflammation. 2014;11:3 Epub 2014/01/09. 10.1186/1742-2094-11-3 1742-2094-11-3 [pii]. 24397957 PMC3895821

[25] Bianchi ME. HMGB1 loves company. J Leukoc Biol. 2009;86(3):573–6. Epub 2009/05/06. 10.1189/jlb.1008585 jlb.1008585 [pii]. . 19414536

[26] Ribes S, Ebert S, Regen T, Agarwal A, Tauber SC, Czesnik D, et al Toll-like receptor stimulation enhances phagocytosis and intracellular killing of nonencapsulated and encapsulated Streptococcus pneumoniae by murine microglia. Infect Immun. 2010;78(2):865–71. Epub 2009/11/26. 10.1128/IAI.01110-09 IAI.01110-09 [pii]. 19933834 PMC2812218

[27] Redlich S, Ribes S, Schutze S, Eiffert H, Nau R. Toll-like receptor stimulation increases phagocytosis of Cryptococcus neoformans by microglial cells. J Neuroinflammation. 2013;10:71 Epub 2013/06/07. 10.1186/1742-2094-10-71 1742-2094-10-71 [pii]. 23738865 PMC3693974

[28] Boche D, Perry VH, Nicoll JA. Review: activation patterns of microglia and their identification in the human brain. Neuropathol Appl Neurobiol. 2013;39(1):3–18. Epub 2012/12/21. 10.1111/nan.12011 . 23252647

[29] Chhor V, Le Charpentier T, Lebon S, Ore MV, Celador IL, Josserand J, et al Characterization of phenotype markers and neuronotoxic potential of polarised primary microglia in vitro. Brain Behav Immun. 2013;32:70–85. Epub 2013/03/05. 10.1016/j.bbi.2013.02.005 S0889-1591(13)00127-X [pii]. 23454862 PMC3694309

[30] Colton CA. Heterogeneity of microglial activation in the innate immune response in the brain. J Neuroimmune Pharmacol. 2009;4(4):399–418. Epub 2009/08/06. 10.1007/s11481-009-9164-4 19655259 PMC2773116

[31] Mantovani A, Sozzani S, Locati M, Allavena P, Sica A. Macrophage polarization: tumor-associated macrophages as a paradigm for polarized M2 mononuclear phagocytes. Trends in immunology. 2002;23(11):549–55. . 12401408 10.1016/s1471-4906(02)02302-5

[32] Kawanaka N, Taylor AW. Localized retinal neuropeptide regulation of macrophage and microglial cell functionality. J Neuroimmunol. 2011;232(1–2):17–25. 10.1016/j.jneuroim.2010.09.025 20965575 PMC3030990

[33] Morris SM Jr. Arginine metabolism: boundaries of our knowledge. The Journal of nutrition. 2007;137(6 Suppl 2):1602S–9S. . 17513435 10.1093/jn/137.6.1602S

[34] Yao L, Kan EM, Lu J, Hao A, Dheen ST, Kaur C, et al Toll-like receptor 4 mediates microglial activation and production of inflammatory mediators in neonatal rat brain following hypoxia: role of TLR4 in hypoxic microglia. J Neuroinflammation. 2013;10:23 10.1186/1742-2094-10-23 23388509 PMC3575244

[35] Zhou H, Lapointe BM, Clark SR, Zbytnuik L, Kubes P. A requirement for microglial TLR4 in leukocyte recruitment into brain in response to lipopolysaccharide. J Immunol. 2006;177(11):8103–10. . 17114485 10.4049/jimmunol.177.11.8103

[36] Tian S, Zhang L, Tang J, Guo X, Dong K, Chen SY. HMGB1 exacerbates renal tubulointerstitial fibrosis through facilitating M1 macrophage phenotype at the early stage of obstructive injury. American journal of physiology Renal physiology. 2015;308(1):F69–75. 10.1152/ajprenal.00484.2014 25377911 PMC4281691

[37] Kawai T, Akira S. Signaling to NF-kappaB by Toll-like receptors. Trends Mol Med. 2007;13(11):460–9. Epub 2007/11/22. S1471-4914(07)00184-0 [pii] 10.1016/j.molmed.2007.09.002 . 18029230

[38] Manna SK, Aggarwal BB. Alpha-melanocyte-stimulating hormone inhibits the nuclear transcription factor NF-kappa B activation induced by various inflammatory agents. J Immunol. 1998;161(6):2873–80. Epub 1998/09/22. . 9743348

[39] Sarkar A, Sreenivasan Y, Manna SK. alpha-Melanocyte-stimulating hormone inhibits lipopolysaccharide-induced biological responses by downregulating CD14 from macrophages. FEBS Lett. 2003;553(3):286–94. Epub 2003/10/24. S0014579303010299 [pii]. . 14572638 10.1016/s0014-5793(03)01029-9

[40] Hilliard BA, Mason N, Xu L, Sun J, Lamhamedi-Cherradi SE, Liou HC, et al Critical roles of c-Rel in autoimmune inflammation and helper T cell differentiation. The Journal of clinical investigation. 2002;110(6):843–50. 10.1172/JCI15254 12235116 PMC151124

[41] Sanjabi S, Hoffmann A, Liou HC, Baltimore D, Smale ST. Selective requirement for c-Rel during IL-12 P40 gene induction in macrophages. Proc Natl Acad Sci U S A. 2000;97(23):12705–10. 10.1073/pnas.230436397 11058167 PMC18828

[42] Watford WT, Moriguchi M, Morinobu A, O'Shea JJ. The biology of IL-12: coordinating innate and adaptive immune responses. Cytokine & growth factor reviews. 2003;14(5):361–8. . 12948519 10.1016/s1359-6101(03)00043-1

[43] Iwakura Y, Ishigame H. The IL-23/IL-17 axis in inflammation. The Journal of clinical investigation. 2006;116(5):1218–22. 10.1172/JCI28508 16670765 PMC1451213

[44] Taylor AW, Kitaichi N. The diminishment of experimental autoimmune encephalomyelitis (EAE) by neuropeptide alpha-melanocyte stimulating hormone (alpha-MSH) therapy. Brain Behav Immun. 2008;22(5):639–46. Epub 2008/01/04. S0889-1591(07)00274-7 [pii] 10.1016/j.bbi.2007.11.001 18171609 PMC3337335

[45] Heyen JR, Ye S, Finck BN, Johnson RW. Interleukin (IL)-10 inhibits IL-6 production in microglia by preventing activation of NF-kappaB. Brain research Molecular brain research. 2000;77(1):138–47. . 10814840 10.1016/s0169-328x(00)00042-5

[46] Dennehy KM, Ferwerda G, Faro-Trindade I, Pyz E, Willment JA, Taylor PR, et al Syk kinase is required for collaborative cytokine production induced through Dectin-1 and Toll-like receptors. Eur J Immunol. 2008;38(2):500–6. 10.1002/eji.200737741 18200499 PMC2430329

[47] de Waal Malefyt R, Abrams J, Bennett B, Figdor CG, de Vries JE. Interleukin 10(IL-10) inhibits cytokine synthesis by human monocytes: an autoregulatory role of IL-10 produced by monocytes. The Journal of experimental medicine. 1991;174(5):1209–20. 1940799 10.1084/jem.174.5.1209 PMC2119001

[48] Matsui T, Tasaki M, Yoshioka T, Motoki Y, Tsuneoka H, Nojima J. Temperature- and time-dependent changes in TLR2-activated microglial NF-kappaB activity and concentrations of inflammatory and anti-inflammatory factors. Intensive care medicine. 2012;38(8):1392–9. 10.1007/s00134-012-2591-3 . 22653369

[49] Funderburg NT, Jadlowsky JK, Lederman MM, Feng Z, Weinberg A, Sieg SF. The Toll-like receptor 1/2 agonists Pam(3) CSK(4) and human beta-defensin-3 differentially induce interleukin-10 and nuclear factor-kappaB signalling patterns in human monocytes. Immunology. 2011;134(2):151–60. 10.1111/j.1365-2567.2011.03475.x 21896010 PMC3194223

[50] Cao S, Zhang X, Edwards JP, Mosser DM. NF-kappaB1 (p50) homodimers differentially regulate pro- and anti-inflammatory cytokines in macrophages. J Biol Chem. 2006;281(36):26041–50. 10.1074/jbc.M602222200 16835236 PMC2642587

[51] Clarke CJ, Hales A, Hunt A, Foxwell BM. IL-10-mediated suppression of TNF-alpha production is independent of its ability to inhibit NF kappa B activity. Eur J Immunol. 1998;28(5):1719–26. . 9603479 10.1002/(SICI)1521-4141(199805)28:05<1719::AID-IMMU1719>3.0.CO;2-Q

[52] Yao J, Mackman N, Edgington TS, Fan ST. Lipopolysaccharide induction of the tumor necrosis factor-alpha promoter in human monocytic cells. Regulation by Egr-1, c-Jun, and NF-kappaB transcription factors. J Biol Chem. 1997;272(28):17795–801. . 9211933 10.1074/jbc.272.28.17795

[53] Udalova IA, Knight JC, Vidal V, Nedospasov SA, Kwiatkowski D. Complex NF-kappaB interactions at the distal tumor necrosis factor promoter region in human monocytes. J Biol Chem. 1998;273(33):21178–86. . 9694874 10.1074/jbc.273.33.21178

[54] Ponomarev ED, Maresz K, Tan Y, Dittel BN. CNS-derived interleukin-4 is essential for the regulation of autoimmune inflammation and induces a state of alternative activation in microglial cells. J Neurosci. 2007;27(40):10714–21. 10.1523/JNEUROSCI.1922-07.2007 . 17913905 PMC6672829

[55] Suzumura A, Sawada M, Itoh Y, Marunouchi T. Interleukin-4 induces proliferation and activation of microglia but suppresses their induction of class II major histocompatibility complex antigen expression. J Neuroimmunol. 1994;53(2):209–18. Epub 1994/09/01. . 8071435 10.1016/0165-5728(94)90031-0 PMC7119647

[56] Pisetsky DS, Erlandsson-Harris H, Andersson U. High-mobility group box protein 1 (HMGB1): an alarmin mediating the pathogenesis of rheumatic disease. Arthritis Res Ther. 2008;10(3):209 Epub 2008/07/05. ar2440 [pii] 10.1186/ar2440 18598385 PMC2483460

[57] Campana L, Bosurgi L, Rovere-Querini P. HMGB1: a two-headed signal regulating tumor progression and immunity. Curr Opin Immunol. 2008;20(5):518–23. Epub 2008/07/05. S0952-7915(08)00054-X [pii] 10.1016/j.coi.2008.04.012 . 18599281

[58] Napoli I, Neumann H. Microglial clearance function in health and disease. Neuroscience. 2009;158(3):1030–8. 10.1016/j.neuroscience.2008.06.046 . 18644426

[59] Neher JJ, Neniskyte U, Zhao JW, Bal-Price A, Tolkovsky AM, Brown GC. Inhibition of microglial phagocytosis is sufficient to prevent inflammatory neuronal death. J Immunol. 2011;186(8):4973–83. Epub 2011/03/16. 10.4049/jimmunol.1003600 jimmunol.1003600 [pii]. . 21402900

[60] Neher JJ, Emmrich JV, Fricker M, Mander PK, Thery C, Brown GC. Phagocytosis executes delayed neuronal death after focal brain ischemia. Proc Natl Acad Sci U S A. 2013;110(43):E4098–107. 10.1073/pnas.1308679110 24101459 PMC3808587

